# Epidemiology of burnout syndrome in four occupational sectors in Cameroon-impact of the practice of physical activities and sport

**DOI:** 10.3934/publichealth.2020027

**Published:** 2020-06-04

**Authors:** J Mekoulou Ndongo, CE Bika Lélé, LJ Owona Manga, PT Moueleu Ngalagou, CN Ayina Ayina, MY Lobe Tanga, WR Guessogo, N Barth, B Bongue, SH Mandengue, LS Etoundi Ngoa, PB Assomo Ndemba

**Affiliations:** 1 Physiology and Medicine of Physical Activities and Sports Unit, University of Douala, Cameroon; 2 Institute of Medicinal Plants, Ministry of Scientific Research and Innovation, Yaounde, Cameroon; 3 Department of Public Health, Faculty of Medicine and Pharmaceutical Sciences, University of Douala, Cameroon; 4 National Institute of Youth and Sport, University of Yaounde I, Cameroon; 5 Laboratoire EA 4607 SNA-EPIS, Université Jean Monnet, 42100 Saint-Etienne, France; 6 Department of Physiology, Faculty of Medicine and Biomedical Sciences, University of Yaounde I, Cameroon

**Keywords:** burnout syndrome, risk factors, physical activities, Cameroon

## Abstract

**Background:**

The purpose of this study was to determine the prevalence of Burnout syndrome (BOS), risk factors and the effect of physical activity in six professions in Cameroon.

**Methods:**

2012 participants completed questionnaires related to socio-demographic conditions and work perception. Appropriate Maslach Burnout Inventory (MBI) psychometers were used for specific professions. Level of physical activity and sports practice was determined using the Ricci and Gagnon scale.

**Results:**

The overall prevalence of burnout was 67.9%; with 5.3% high; 34.3% moderate; and 60.4% low degree. 42.2% of victims of BOS were in high loss of personal achievement, 39.9% in high depersonalization of and 38.2% in high emotional exhaustion. Higher prevalence of BOS was found in Army (85.3%) and educational sectors (78.5% in secondary school teachers (SET) and 68% in university teaching staff (UTS)). BOS was significantly associated (p < 0.05) with distance from home to workplace, number of children per participant, number of hospitals attended, number of guards per month, labour hours per day, conflicts with the hierarchy, conflicts with colleagues, poor working conditions, unsatisfactory salary, part time teaching in private university institutions, job seniority, sedentariness. Apart from UTS, no association was observed between the level of physical activity and occurrence of BOS.

**Conclusion:**

Burnout is a reality in occupational environments in Cameroon.

## 1. Introduction

All over the world, workers are facing high occupational deregulation, job insecurity, conflicting conditions, high workload, and job-stress linked to the demand of high standards, quality services, and high competition [1]. This situation leads to overwork and exposes workers to a state of exhaustion commonly known as Burnout syndrome, conceptualized for the first time by Freudenberg in 1974. This concept was later refined as an affection of three dimensions: emotional exhaustion (EE), depersonalisation or dehumanisation (DP) and loss of personal accomplishment (LPA) [2].

Burnout syndrome is a psychosocial phenomenon which arises as a response to chronic interpersonal stressors. It is a global concern and work-related stress has the potential to negatively affect psychological and physical health, as well as organization effectiveness. Chronic stress exposure is the first key factor in the causality of burnout. Second, negative coping strategies contribute to the maintenance and aggravation of burnout symptoms. Third, on the physiopathological aspect, it's known that both stress and burnout have been linked with a dysregulation of the hypothalamic-pituitary-adrenocortical (HPA) axis [3]. Burnout was found associated with poor health, including some non-communicable diseases [4],[5]. While initially involved in healthcare and education profession, burnout gradually expanded to include almost all occupational sectors [6],[7].

According to the World Health Organization (WHO) [8], exhaustion is considered a major problem in workplaces with a prevalence of 20% in active populations. However, the clinical symptoms are not closely related to burnout; and this is a major problem of diagnosis to medical practitioners.

For many years, BOS was not considered formally as a disease in the International Classification of Diseases (ICD) of the WHO, nor by the Diagnostic and Statistical Manual (DSM) of the American Psychiatric Association. Recently, in the 10th edition of the International Classification of Diseases (ICD-10) code Z73.0, burnout has been identified as a factor influencing health status and help-seeking with health services and coded Z73.0 and defined as a state of vital exhaustion (ICD-10).

The prevention of burnout requires the improvement of working conditions, adjustment of workloads and regular practice of physical activities and sports. Some studies have reported therapeutic and preventive effects of physical activities and sport against burnout [9]–[11], cardiovascular diseases, diabetes, cognitive functions, metabolic syndrome, aging, cancers, stress, and depression [12],[13].

Cameroon is a developing country where some modern society conditions related to job environment and international standards are not respected. This is apparent in several occupational sectors such as health, education, defense and security forces, among others. However, there is a real mismatch between job demands and job resources which makes working conditions constraints by increasing the chronic exposure to stress, which is known to be the main cause of human exhaustion.

To our knowledge, before 2015, no study has been carried out on burnout syndrome in Cameroon. The present study is the round-up of a project put in place in Cameroon in 2015 with the general purpose of investigating the epidemiology of burnout syndrome on a variety of professions and subsequently evaluate the eventual impact of the practice of physical activity to prevent it.

## 2. Methods

### 2.1. Design of the study

The present study was a multi-centric cross-sectional investigation, conducted in Cameroon from 2015 to 2018. We focused on four occupational sectors:

1. Healthcare sector; including general medical doctors (GMD), specialist medical doctors (SMD) and paramedical staff (PMS);
2. Education sector; including secondary education teacher (SET) and university teaching staff (UTS);
3. Private sector workers (PSW);
4. Army;

We focused on these occupational sectors because, on one hand, they are sectors of human services, and on the other, there is an important exposure to stressors related to human contacts emphasizing emotional load. Pregnant women, persons with documented pathology, such as cognitives and infectious diseases were excluded.

Participants were recruited using a non-probabilistic voluntary enrolment. Detailed explanation was given on the aim of methodology to each participant before giving a free consent to participate. This study was approved by the Institutional Ethics Committee of the University of Douala under the reference number: CEI-UD/389/01/2016/T and was conducted in conformity with the recommendations of the Declaration of Helsinki revised in 1989. A total of 2012 participants were voluntarily included, and the sample was distributed in 302 PMS, 85 GMD, 330 SET, 209 SMD, 424 PWS and 354 Army personnel.

### 2.2. Instruments and data collection

Data were collected using a self-administered questionnaires consisting in five main sections:

(i). *Sociodemographic characteristics:* age, sex, marital status, number of dependent children.

(ii). *Social-professional and work conditions:* speciality, seniority, working conditions, labour hours per week, number of free weekends per month, number of vacation days per year, perception of labour hardship, conflicts with colleagues and hierarchy, job salary satisfaction.

(iii). *Level of physical activity:* determined using the questionnaire of Ricci and Gagnon [14]. This questionnaire is a scale divided in two sub-sections, A and B, each with four items. Sub-section A evaluates the duration and intensity of daily common activities such as cleaning, gardening, rural work, walking. Sub-section B evaluates sport and recreational activities. The total of points in sub-sections A and B classify participants as inactive if the score < 16, active between 16 and 32, very active if the score > 32.

(iv). *Maslach Burnout Inventory (MBI):* appropriate and specific for each occupational sector as follows:

a. *MBI-Human Services Survey* (*MBI-HSS*), was used among health professionals (PMP, GMD and SMD). This psychometric instrument consists of 22 items and explores the three dimensions of burnout, which are:

- EE with 9 items: 1, 2, 3, 6, 8, 13, 14, 16, 20;
- DP with 5 items: 5, 10, 11, 15, 22;
- LPA with 8 items: 4, 7, 9, 12, 17, 18, 19, 21;

The scores in each dimension are allowed to characterize the level of BOS as high, moderate or low.

b. *MBI-General Survey (MBI-GS)*, was used to assess BOS in Army and private sector employees. This psychometric consists of sixteen items and three scales that refer to the three dimensions of burnout syndrome:

- EE or Exhaustion: 5 items (1, 2, 3, 4 and 6);
- DP or dehumanisation of the relationship or cynicism: 5 items (8, 9, 13, 14, 15);
- LAP: 6 items (5, 7, 10, 11, 12, and 16);

c. *MBI-Educators Survey (MBI-ES)* was used for SET and UTS. MBI-ES includes 22 items that determine the three dimensions of burnout:

- EE (9 items): 1, 2, 3, 6, 8, 13, 14, 16, 20;
- DP (5 items): 5, 10, 11, 15, 22;
- LPA (8 items): 4, 7, 9, 12, 17, 18, 19, 21;

d. The response modalities for the 22 items are based on a 7-point frequency with an intensity from 0 to 6: “0 = never” to “6 = every day”. The score for each dimension is used to determine level of affect as high, or as low:

- EE: high ≥ 27; 17–26 low ≤ 16;
- DP: high ≥ 13; 7–12 low ≤ 6;
- LPA: high ≤ 30; 31–36 low ≥ 37.

Questionnaires were handed to participants for one week at least, to take cognition of the content and give enough time to answer the various items.

(v). *Clinical symptoms associated with BOS and job perception:* a questionnaire was developed to identify some clinical symptoms in order to see if they were associated with BOS in affected participants.

### 2.3. Statistical analysis

Data were recorded and processed using the EPI Info 7 and Excel 2007 software and analysed using the XL Stat 7.5.2 software. Quantitative and qualitative variables are presented as mean ± standard deviation (SD) and frequency associated with percentages respectively. In two-way analysis, the qualitative variables were compared using the Chi 2 test and the exact Fisher probability was determined for dichotomous variables. The raw Odds Ratio (OR) and their confidence interval were determined. For the comparison of quantitative variables, students' t-tests were used. In multivariate analysis, the logistic regression test was used to establish the relationship between burnout and the previously associated predictor variables in univariate. Results were considered significant for p < 0.05.

## 3. Results

Socio-demographic characteristics and physical activity characteristics, professional and psychosocial description of participants are presented in Table 1.

**Table 1.** Socio-demographic, physical activity, professional and psychosocial description of participants.

		All (2012)	SET (330)	PSW (424)	PMS (302)	GMD (85)	UTS (303)	SMD (209)	Army (354)	p
Age (years)	m ±SD	36 ±9	38 ±8	40 ±9	37 ±7	29 ±4	43 ±7	30 ±3	33 ±8	<0.001
Gender	F	36.4	54.6	18.2	67.2	48.2	31.0	47.4	9.9	
	M	63.6	45.4	81.8	32.8	51.8	69.0	52.6	90.1	<0.001
Married	No	52.0	27.8	74.1	44.7	75.3	25.1	64.1	65.0	
	Yes	48.0	72.2	25.9	55.3	24.7	74.9	35.9	35.0	<0.001
Children in charge	0	32.4	19.7	54.5	15.9	60.0	11.6	50.2	32.5	
	1	14.7	12.5	9.2	21.5	28.2	12.2	19.1	13.8	
	2–4	41.5	51.9	26.2	53.3	11.8	67.7	30.1	31.1	
	5	11.5	15.8	10.1	9.3	0.0	8.6	1.0	22.6	<0.001
Physical activity	Yes	63.6	65.1	59.1	74.8	51.8	46.5	42.6	85.6	
	No	36.4	34.9	40.9	25.2	48.2	53.5	57.4	14.4	<0.001
Job seniority (years)	< 5	33.9	16.4	35.8	25.8	80.0	25.1	64.1	33.6	
	5–10	35.2	46.3	28.5	36.4	18.8	48.2	35.4	24.3	
	> 10	30.9	37.3	35.6	37.7	1.2	26.7	0.5	42.1	<0.001
Working time (hrs/day)	< 8	37.5	81.2	0.4	0.3	18.8	75.6	71.3	5.1	
	8	30.9	17.6	74.0	20.2	31.8	23.8	28.7	30.5	
	> 8	31.6	1.2	25.6	79.5	49.4	0.7	0.0	64.4	<0.001
Secondary work	No	61.7	48.7	71.9	55.6	56.5	18.2	99.0	88.7	
	Yes	38.3	51.3	28.1	44.4	43.5	81.8	1.0	11.3	<0.001
Salary satisfaction	No	76.5	93.4	51.4	72.8	83.5	99.0	90.0	65.0	
	Yes	11.0	6.6	4.5	19.5	16.5	1.0	3.3	27.7	<0.001
Work exhausting	No	51.3	39.1	66.1	26.8	57.6	60.4	44.0	68.6	
	Yes	48.7	60.9	33.9	73.2	42.4	39.6	56.0	31.4	<0.001
Work constraining	No	69.7	63.9	90.1	71.5	58.8	62.4	56.0	76.6	
	Yes	30.3	36.1	9.9	28.5	41.2	37.6	44.0	23.4	<0.001
Work laborious	No	77.3	72.8	83.9	64.9	88.2	70.3	85.6	85.9	
	Yes	22.7	27.2	16.1	35.1	11.8	29.7	14.4	14.1	<0.001
Work stressing	No	63.4	61.5	66.5	59.3	52.9	65.3	40.2	81.4	
	Yes	36.6	38.5	33.5	40.7	47.1	34.7	59.8	18.6	<0.001
No work complaint	No	86.0	90.7	81.0	90.1	87.1	85.5	91.4	78.2	
	Yes	14.0	9.3	19.0	9.9	12.9	14.5	8.6	21.8	<0.001
Conflict with hierarchy	No	83.7	83.6	86.4	91.7	83.5	76.6	71.8	88.1	
	Yes	16.3	16.4	13.6	8.3	16.5	23.4	28.2	11.9	<0.001
Conflicts with colleagues	No	80.7	76.1	86.0	74.5	84.7	78.5	78.0	89.0	
	Yes	19.3	23.9	14.0	25.5	15.3	21.5	22.0	11.0	<0.001
Need of job conversion	No	62.7	55.5	41.3	54.0	75.3	75.2	51.7	84.2	
	Yes	37.3	44.5	58.7	46.0	24.7	24.8	48.3	15.8	<0.001

Note: GMD: Generalist medical doctor, PMS: Paramedical staff; PSW: Private sector worker; SET: Secondary education teacher; SMD: specialist medical doctor; UTS: University teaching staff; BOS: Burnout syndrome.

The mean age of the overall population was 36 ± 9 years, and 63.6% of participants were male. Males were predominant in PSW, GMD, UTS, SMD and Army. More than a half (52%) of the participants were unmarried. 41.5% of participants had 2–4 children. The majority of the participants (63.6%) practiced physical activities, mostly in Army followed by PMS and SET. On the other hand, SMD and UTS practiced fewer physical activities.

**Figure 1.**
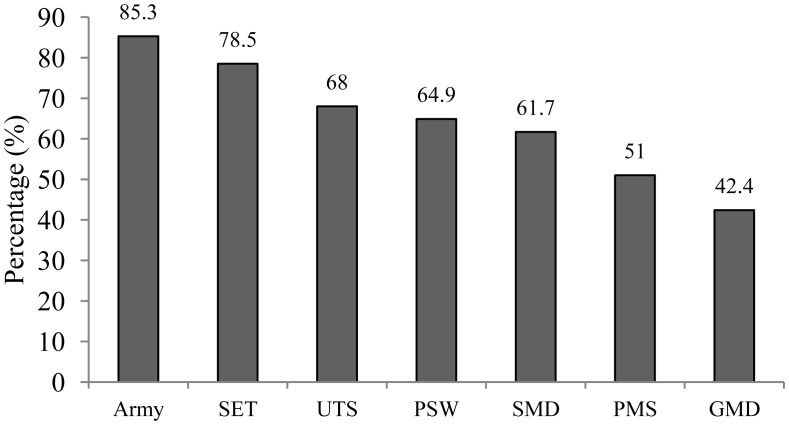
Prevalence of BOS in each group. GMD: Generalist Medical Doctor; PMS: Paramedical Staff; PSW: Private Sector Worker; SET: Secondary Education Teacher; SMD: Specialist Medical Doctor; UTS: University Teaching Staff; BOS: Burnout syndrome.

**Figure 2.**
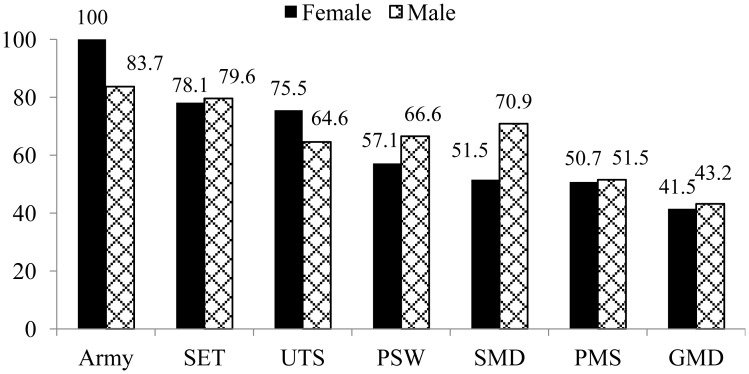
Prevalence of BOS according to gender. GMD: Generalist Medical Doctor; PMS: Paramedical Staff; PSW: Private Sector Worker; SET: Secondary Education Teacher; SMD: Specialist Medical Doctor; UTS: University Teaching Staff; BOS: Burnout syndrome.

The prevalence of BOS by profession and the distribution related to gender are shown in Figure 1 and 2:

Burnout was highly present in Army with a prevalence of 85.3% followed SET (78.5%) and UTS (68%). More than a half of PSW (64.4%), SMD (61.7%) and PMS (51%) were affected by BOS, while BOS was less present in GMD (42.4%). Except in Army and UTS, men were more victims of BOS compared to women.

**Table 2.** Prevalence, level and different components of BOS in the sample compared between male and female.

			Total n (%)		Female n (%)		Male n (%)		P
BOS	No		646 (32.1)		268 (36.6)		378 (29.5)		0.001
	Yes		1366 (67.9)		464 (63.4)		902 (70.5)		
Level of BOS*	Low		825 (60.4)		284 (61.2)		541 (60.0)		0.792
	Moderate		469 (34.3)		158 (34.1)		311 (34.5)		
	High		72 (5.3)		22 (4.7)		50 (5.5)		
LPA	Low		707 (35.1)		296 (40.4)		411 (32.1)		< 0.001
	Moderate		456 (22.7)		188 (25.7)		268 (20.9)		
	High		849 (42.2)		248 (33.9)		601 (47.0)		
DP	Low		802 (39.9)		337 (46.0)		465 (36.3)		< 0.001
	Moderate		660 (32.8)		236 (32.2)		424 (33.1)		
	High		550 (27.3)		159 (21.7)		391 (30.5)		
EE	Low		514 (25.5)		172 (23.5)		342 (26.7)		0.002
	Moderate		729 (36.2)		302 (41.3)		427 (33.4)		
	High		769 (38.2)		258 (35.2)		511 (39.9)		
		Army (%)	SET (%)	UTS (%)	PSW (%)	SMD (%)	PMS (%)	GMD (%)	
LPA	Low	29.4	40.6	54.1	21.5	28.2	31.1	69.4	<0.0001
	Moderate	20.9	21.8	23.8	13.9	27.8	34.4	18.8	
	High	49.7	37.6	22.1	64.6	44.0	34.4	11.8	
DP	Low	7.6	54.0	39.6	49.1	36.4	46.7	57.6	<0.0001
	Moderate	32.8	29.6	28.1	28.1	44.5	40.1	31.8	
	High	59.6	16.4	32.3	22.9	19.1	13.2	10.6	
EE	Low	24.0	20.6	31.4	27.8	29.7	16.9	40.0	<0.0001
	Moderate	26.8	26.0	53.5	27.8	27.8	60.9	29.4	
	High	49.2	53.4	15.2	44.3	42.6	22.2	30.6	

Note: GMD: Generalist Medical Doctor; PMS: Paramedical Staff; PSW: Private Sector Worker; SET: Secondary Education Teacher; SMD: Specialist Medical Doctor; UTS: University Teaching Staff; BOS: Burnout syndrome.

High LPA was significantly (P < 0.0001) observed in GMD (69.4%) and moderate in PSW (64.6%). Low DP was more in GMD than other professions. However, high DP was significantly (P < 0.0001) present in Army (59.6%). Indeed, moderate EE was significantly (P < 0.0001) higher in PMS (60.9%) and UTS (53.5%) than others' professions.

### 3.1. Clinical symptoms associated with BOS

The top ten clinical symptoms found among participants who suffered from BOS in the sample were: asthenia (53%), back pain (41%), headache (38%), mistrust (27%), irritability (25%), hyperactivity (22%), hypersensitivity (19%), fit of anger (16%), stomachache (12%), and palpitations (8%).

**Table 3.** Factors associated to BOS in the whole population.

		%	OR (95%CI)	p	AOR* (95%CI)	p
Age (years)	< 30	70.4	1		1	
	≥ 30	68.4	0.91 (0.72–1.15)	0.420	0.92 (0.73–1.16)	0.478
Gender	F	64.0	1		1	
	M	71.8	1.43 (1.17–1.75)	0.001	1.42 (1.17–1.86)	0.001
Married	No	68.5	1		1	
	Yes	69.2	1.03 (0.84–1.26)	0.770	1.11 (0.89–1.39)	0.338
Number children	0	68.9	1		1	
	1	67.9	0.96 (0.70–1.31)	0.780	1.03 (0.75–1.42)	0.843
	2–4	67.3	0.93 (0.73–1.18)	0.552	1.03 (0.78–1.36)	0.825
	5	75.8	1.41 (1.01–2.02)	0.045	1.49 (1.01–2.20)	0.042
Job seniority (years)	< 5	66.4	1		1	
	5–10	69.9	1.17 (0.92–1.49)	0.195	1.33 (1.02–1.74)	0.037
	> 10	70.2	1.19 (0.93–1.52)	0.172	1.33 (1.00–1.78)	0.043
Work time (hrs/day)	< 8	70.7	1		1	
	8	68.7	0.91 (0.71–1.16)	0.439	0.86 (0.67–1.10)	0.231
	> 8	66.8	0.83 (0.65–1.05)	0.124	0.79 (0.62–1.01)	0.057
Secondary work	No	69.9	1		1	
	Yes	67.2	0.88 (0.72–1.08)	0.226	0.89 (0.72–1.10)	0.280
Salary satisfaction	No	69.5	1		1	
	Yes	64.0	0.78 (0.58–1.04)	0.094	0.76 (0.57–1.03)	0.076
Work exhausting	No	67.9	1		1	
	Yes	69.8	1.09 (0.89–1.33)	0.389	1.12 (0.92–1.37)	0.256
Work constraining	No	66.7	1		1	
	Yes	73.9	1.41 (1.13–1.77)	0.002	1.43 (1.14–1.79)	0.002
Work laborious	No	66.4	1		1	
	Yes	77.2	1.71 (1.33–2.20)	<0.001	1.77 (1.37–2.29)	<0.001
Work stressing	No	66.1	1		1	
	Yes	73.5	1.42 (1.15–1.76)	0.001	1.47 (1.19–1.82)	<0.001
No work complaint	No	70.6	1		1	
	Yes	58.4	0.58 (0.45–0.77)	<0.001	0.57 (0.44–0.75)	<0.0001
Conflict with hierarchy	No	67.3	1		1	
	Yes	76.6	1.59 (1.19–2.12)	0.002	1.58 (1.18–2.11)	0.002
Need of job conversion	No	67.6	1		1	
	Yes	71.0	1.18 (0.96–1.45)	0.124	1.21 (0.98–1.48)	0.078
Physical activity	No	69.5	1		1	
	Yes	68.5	0.90 (0.73–1.11)	0.343	0.95 (0.77–1.17)	0.641

Note: OR: odd ratio; AOR: adjusted odd ratio (*adjusted for age and sex); n (%) represent number and percentage of participants with BOS.

Except in UTS (OR = 0.51; 95% CI (0.31–0.84); P = 0.009), we did not find a statistical difference between the practice of sporting activities and BOS.

## 4. Discussion

This study aimed to determine the prevalence of burnout, associated factors, and to assess the influence of physical activities on this psychopathology in four occupational sectors, including health, education, Army and the private sector.

This study is the first conducted in Cameroon including several professional sectors. Known studies conducted in Cameroon on burnout mostly gave scores on the OLBI scale without precision on prevalence and were only concerned with medical students and health professionals [15]–[18].

Since participants were recruited using a non-probabilistic voluntary enrolment, the large size sample of our study aimed to minimize the lack of representativeness versus the average national workforce estimated to be 264535 workers in 2015 [19] and to increase the significance and validity of our statistical analyses.

An overall prevalence of 67.9% of BOS was found. This result is similar and consistent with other studies that focused specifically on a single occupational group [20],[21]. The high prevalence observed could be justified either by the limits of the methodological approach (cross sectional study and limits of BOS definition) or the difficult socioeconomic context of workers in Cameroon. In fact, the socioeconomic condition of civil servants' salaries are very difficult considering the CFA Franc devaluation in 1994 and the multiple socioeconomic adjustment programs of the nineties. All these conditions are permanent sources of dissatisfaction, low motivation, attrition, and chronic stress, which are predictors of BOS.

We found in general, except in Army and in UTS, men were more affected by BOS than women (70.5% vs. 63.4%). This global result on burnout and gender is controversial to those authors [22],[23] who found high prevalence of burnout in women. According to Norlund et al. [22], a high level of burnout in women compared to men was partly explained by more unfavourable working conditions and life situational factors. Our finding of high prevalence of BOS among men compared to women is in accordance with Tironi et al. [24].

A prevalence of 85.3% of BOS found in the Cameroonian Army was higher than in the other professions and was similar to the 80.1% reported by de Tao et al. [25] among soldiers from drylands in China. This result is higher than the one found by Pearson et al. [26] among Canadian soldiers (13.5–48.4%) and among the Senegalese Army (39.9%) returning from a mission in Darfur [27]. The high prevalence of burnout in the Cameroonian Army can be explained by the high workload of soldiers who are permanently exposed to the stress related to battles against the Boko Haram militia and the internal socio-political situation of the north-west and the south-west regions. Furthermore, some Cameroonian soldiers carry out peacekeeping missions in Central Africa Republic. According to Morgan et al. [28], soldiers, during their service, spend considerable time under stress-intensive conditions with the population in terms of performance requirements and the intensity of the situations; generating frustrations, anger, and the feeling of being without support. When these feelings are experienced for long periods, they constitute appropriate conditions for the occurrence of burnout. The prevalence of BOS (78.8%) found among SET was higher than the one reported by Restrepo et al. [29] in Columbia and Chennoufi et al. [30] in Tunisia; whereas the prevalence found among UTS (68%) was lower than the 74% reported by Lokanadha and Poornima [31], in India. The higher prevalence of BOS among SET compared to UTS in our study is in contrast with the study of Farber [32] which observed that university professors are at greater risk of burnout than those in high school because of work overload. Our results are in accordance with Anderson et al. [33], who found that high school teachers are more at risk of burnout than primary school and university teachers. The difference in the present study can be explained by the number of hours per week and workload of the secondary school teachers which is higher than university teachers. Moreover, the difference in the ratio of schoolchildren per teacher was higher in secondary schools compared to university. The high prevalence of BOS in education sectors (SET and UTS) could also be explained by Cameroon's failure to meet UNESCO standards in terms of the ratio of learners per teacher, which is two to three times superior, thus increasing the workload and predisposing to BOS. According to Van der Broech et al. [34], the risk of BOS may be explained by the combination of a high level of job applications and job resources in a given activity. Demerouti et al. [30] also showed in their “Job Demands Resources” concept that demands exhaust workers' resources and thus increase burnout, while resources diminish the sense of exhaustion. There are some factors that can contribute to the development of burnout (job requests) and others that can create enthusiasm (job resources), as these factors are very specific to each job and job situation in general [35].

The prevalence of BOS among PSW (64.9%) was superior to the one in Western countries' workers (13–27%) [36] and in United States (30–33%) [37] and inferior to the one reported by Valente et al. [38] in Brazil (71.8%). Burnout prevalence among GMD (42.4%) was lower than that observed in Egypt (66.7%) by Farahat et al. [39] in the same professional group, whereas the prevalence among SMD (61.7%) was higher than that among British surgeons (32%), as reported by Sharma et al. [20] and among US surgeons (34.6%) as found by Oskrochi et al. [40]. On the other hand, the higher prevalence of BOS in SMD compared to GMD may be explained by the expression of chronic stress, because of previous experience as GMD. The prevalence of BOS among PMS (51%), was greatly lower than that in Tunisia (70%) as reported by Amamou et al. [41] among palliative care nurses and in Nigeria (75%) among caregivers [42].

Low burnout (60.4%), was predominantly observed in the overall population. This shows an insidious and slow installation of BOS among participants, due to permanent exposure to stress and associated factors. Levels of BOS didn't vary significantly between male and female workers.

Among participants with BOS, 38.2% had high EE; 27.3% high DP, and 42.2% a high LAP. These results on BOS dimensions are in accordance with Schaufeli and Enzmann [43] who found that an affection of a specific dimension of the burnout is experienced differently depending on the characteristics of the profession. The severity of the levels of these dimensions is related to stress resulting in a greater exposure of EE, linked to the threat of identity, and linked to the two entities exposed to DP [44].

Low LPA was more observed in GMD (69.4%). This result is in accordance with Soler et al. [45] in Turkish general practitioners. Low LPA in our study is superior than the one observed in others' studies [39],[46]. For Maslash and Jackson [2] a personal achievement decrease represents the dimension of self-evaluation of professional exhaustion related to feelings of incompetence and lack of achievement and productivity at work. This dimension includes both the social and non-social aspects of professional achievement [47]. More specifically, professional effectiveness refers to feelings of competence and success at work. This lack of professional efficiency is the third element of the professional exhaustion building [48].

High DP occurred more in Army (59.6%) than in the other occupational sectors. This result is higher than to those reported by some authors [25],[27]. Depersonalisation represents a negative response, a detachment to various aspects of work, the dimension of the interpersonal context of work exhaustion. Also considered cynical, it reflects indifference, disengagement, lack of enthusiasm, or a distant attitude towards work in general and not necessarily with others.

High EE was noted more in SET (53.4%) than other professions. Maslash et al. [49] in a comparison of the prevalence of BOS between five different occupational sectors in the USA and Holland, found that teaching was characterized by the highest level of exhaustion, along with teachers' cynicism and close to population averages. For Sichambo et al. [50], this level of EE among education staff could be explained by the fact that teachers are exposed to classrooms with a high number of learners, beyond standards, coupling to some duties: examiners, administrators, and follow up with students outside of lectures. According to Roelofs et al. [51], EE can be considered as the most important dimension of BOS because it is close in relationship to other types of mental illness, such as depression and anxiety disorders. Emotional exhaustion refers to the feelings of being emotionally over-extended and drained by others. Nevertheless, BOS is usually considered an individual experience that is specific to the work context.

Our study found that the males were more at risk to be victims of BOS (OR = 1.43, 95% CI (1.17, 1.86), p = 0.001). Despite the under representativeness of women in the study, gender is not considered a strong predictor of burnout according to Maslach et al. [49]. Cathébras et al. [52] observed the inverse relationship where the female gender was at higher risk of BOS. Results on burnout and gender are controversial. Some authors found a higher prevalence of burnout among women and suggested that this was due to their physical and psychological vulnerability to conciliate work and family life [23]. Inversely, others reported a higher rate of burnout among men because of the high workload [24].

Our study found that BOS was associated with the number of children in their charge (OR = 1.49; 95% CI (1.01; 2.20); p = 0.042). This is in accordance with Shanafelt et al. [53]. However, a couple of studies concluded that there was no significant relationship between dependent children and burnout [54],[55], and another found that parental status emerges as a factor that reduces the risk of occupational exhaustion [56]. Malasch et al. [44] also stated that unmarried men tend to be more affected than their colleagues who are married with a higher social charge.

The professional tenure from 5 years and above was a significant determinant of the occurrence of burnout in the present study (OR = 1.33; 95% CI (1.00; 1.78); p = 0.043). This is earlier than what was reported by Teixeira et al. [18] in Portuguese Intensive Care Units who found that previous studies showed that conflicts with colleagues induced emotional exhaustion [57] and led to a reduction in personal achievement, which are two dimensions of BOS. Schaufeli [47] argued that stressful relationships and lack of reciprocity may affect both team workers relationships and the institution and induce burnout. The absence of complaints showed a protective effect against BOS (OR = 0.57; 95% CI (0.44; 0.75); p < 0.0001), showing that satisfactory work conditions and good relationships among working team members can prevent BOS.

Our study found that asthenia, headache, back pain, irritability, mistrust, hypersensitivity, hyperactivity, inclination to anger, stomach aches and palpitations were associated to BOS. A couple of previous studies noted the same signs and symptoms [58],[59],[39]. Though these symptoms may all not be exclusive to BOS, our study shows that their frequency is important to help as guidance to physicians in the diagnosis of BOS in the context of occupational patients. The associated clinical symptoms include fatigue, anxiety, irritability, desire to change jobs, stomach aches, digestion problems, headaches, isolation. In contrast, Embriaco et al. [60] reported that systemic and body complaints: headaches, insomnia and other sleep disorders, diet problems, tiredness, irritability, emotional instability, and rigidity in social relationships are some non-specific symptoms associated with BOS. Local evidence reports that shift work, which was practised by some participants, could also impact the quality of sleep and the social life of health workers in Cameroon [61]. But these factors were not considered during the design of the present study.

The best approach to cope with burnout is to investigate the personality traits of affected individuals, in order to better understand their problem and propose adequate solutions. In fact, Maslach et al. [44] suggested that burnout is linked to a diversity of personality profiles such as the dimension of neuroticism, poor-self-esteem, type-A behaviour, and individuals with “feeling types”.

Assessing the impact of burnout among the affected people is recommended when possible. This can be done through the use of several biomarkers such as cortisol, blood pressure, Dehydroepiandrosterone sulfate (DHEAS), prolactin, the natural killer cells (NK cells) and C-Reactive protein (CRP) that have already been studied in people with burnout [62].

Concerning the strategies to prevent and manage burnout, anticipation is the key. There are multiple strategies, but in our context, we must focus on improving the general environment, the working conditions (salaries) and if possible, construct dedicated areas for practice of sports and physical activities at work as recommended by the WHO [8].

Except in UTS (OR = 0.51; 95% CI (0.31–0.84); P = 0.009), we did not find a statistical difference between the practice of sporting activities and the presence of a syndrome of burnout. Further interventional randomized study is needed to clarify this observation. However, previous studies suggested some beneficial effects of physical activity on reducing stress, depression and burnout [9]–[11]. Gerber et al. [63] found that the enhancement of cardiovascular capacity is associated with high reduction of BOS and depression symptoms. Also, in a study on the effect of a 6-week training program (low intensity jogging 3 times a week) on three indicators of stress-related fatigue (emotional fatigue, general fatigue and recovery need), sleep quality and cognitive functioning in a group of 99 students, compared to the control group, De Vries et al. [64] found that the practice of aerobic exercises has good results on treatment and prevention. This result of the practice of physical activities and sports on burnout in the present study should also draw the attention on the type of physical activity practiced by the participants in this study. In fact, aerobic exercises are known to increase cardiorespiratory capacity and vagal tone and have a positive impact on BOS and depression reduction. In our study we didn't evaluate the cardiorespiratory capacity of participants. Level of practice of physical activities was obtained using a questionnaire. This study also confirms what was previously suggested by Mandengue et al. [58], that BOS could be viewed as a vicious somato-psycho-somatic affection that begins with physical impairment (physical exhaustion), accentuated by socio-demographic environmental factors such as number of dependent children, distance to workplace; and when the body is physically, excessively exhausted, the mind is affected, and in turn, this leads the body to burnout.

The lack of association between physical and sports activities and burnout found among GMD, SMD, PMP, Army, SET and PSW could also be related to the subjective nature (self-reported responses) of the assessment of participants' level of physical activity that is biased. The protective effect of sports and physical activities on the burnout syndrome among UTS was also accompanied by the lowest level of emotional exhaustion (EE) (15.2%). One can view LPA that corresponds to the decrease in self-esteem, and the feeling of being incompetent which are a consequence of the two other dimensions of burnout (emotional exhaustion and depersonalization) negatively impacts the motivation of individuals to be involved in the practice of sports and physical activities, as also observed by Zamani et al. [65].

## 5. Conclusions

1. Burnout syndrome is a reality in full expansion in main national occupations in Cameroon.

2. Burnout syndrome is attributed to several factors, including gender, number of dependent children, stressful constraints, hard work and conflicting relationships at different degrees depending on the occupation.

3. Except in UTS, this study did not find an association between the practice of sporting activities and the presence of symptoms of burnout. Further interventional randomized study is needed to clarify this observation.

4. This study provides the leading main symptoms that are recommended by the World Health Organisation for the diagnosis of BOS in the context of occupational patients.

5. Further studies are needed to study the impact of quality sleep in association with BOS, and also with regard to biomarkers like blood and saliva cortisol, adrenalin, oxytocin, C-RP, and glycemia to understand biological and physiological mechanisms underlying BOS beside epidemiological statistical analysis.
